# Reference Range of Red Cell Distribution Width‐to‐Albumin Ratio in Healthy US Adults: A Cross‐Sectional Study

**DOI:** 10.1155/bmri/9956220

**Published:** 2026-06-04

**Authors:** Parham Habibzadeh, Dennis Hsu

**Affiliations:** ^1^ Department of Medicine, University of Pittsburgh Medical Center, Pittsburgh, Pennsylvania, USA, upmc.com; ^2^ School of Computing and Information, University of Pittsburgh, Pittsburgh, Pennsylvania, USA, pitt.edu; ^3^ UPMC Hillman Cancer Center, Division of Malignant Hematology and Oncology, University of Pittsburgh, Pittsburgh, Pennsylvania, USA, pitt.edu; ^4^ The Ohio State University Comprehensive Cancer Center–Arthur G. James Cancer Hospital and Richard J. Solove Research Institute, Columbus, Ohio, USA

**Keywords:** biomarkers, erythrocyte indices, reference values, risk assessment, serum albumin

## Abstract

The red blood cell distribution width‐to‐albumin ratio (RAR) has been identified as a reliable prognostic marker for all‐cause mortality and various diseases. However, the reference range for RAR across different sexes, age groups, and races remains unknown. This study was conducted to establish age‐, sex‐, and race‐specific reference intervals for the RAR in the general US adult population. A cross‐sectional analysis was performed using seven biennial cycles (2005–2018) of the National Health and Nutrition Examination Survey (NHANES), comprising 22,582 adults aged ≥ 18 years. Pregnant or lactating women and individuals with a history of diabetes mellitus, cardiovascular or cerebrovascular disease, liver disease, thyroid disease, kidney disease, or malignancy were excluded. Age‐, sex‐, and race‐stratified reference intervals (2.5th–97.5th percentiles) for RAR were derived using survey‐weighted methods. The weighted analysis represented more than 141 million US adults. The median RAR was 3.0 %dL/g (IQR: 2.8–3.2), with slightly higher values observed in women compared with men and a modest age‐dependent increase observed. Non‐Hispanic Black individuals were found to have marginally elevated RAR compared with other racial/ethnic groups, along with greater variability. The overall reference interval was determined to be 2.5–4.1 %dL/g. The widest interval was observed among non‐Hispanic Black women aged < 65 years; the narrowest, among Hispanic and non‐Hispanic White men in the same age group. In conclusion, RAR reference intervals showed minimal variation across demographic strata, supporting the potential utility of RAR as a robust biomarker for clinical and research applications.

## 1. Introduction

Identifying new biomarkers for the early detection of individuals at an increased risk of mortality is of paramount importance. Red blood cell distribution width (RDW), an index readily obtainable from routine blood tests, reflects the degree of heterogeneity in red blood cell corpuscular volume. Elevated RDW, reflecting impaired erythropoiesis and reduced survival of abnormal erythrocytes, has been associated with various disease conditions and increased mortality [1].

Serum albumin is also an important marker that reflects the nutritional status and inflammatory response of the body [2, 3]. It exhibits anti‐inflammatory, antioxidant, anticoagulant, and antiplatelet aggregation activities [4]. Several reports have shown that serum albumin levels are inversely associated with the incidence of various disease conditions and increased mortality [5–7].

Not surprisingly, the ratio of RDW to serum albumin concentration (RAR) has been hypothesized to serve as a reliable predictor of mortality in patients with a wide range of diseases. A large cohort study based on data from the US National Health and Nutrition Examination Survey (NHANES) and the UK Biobank reports a higher risk of all‐cause and cause‐specific mortality among those with elevated baseline RAR [8]. Another cohort study, also utilizing NHANES data, shows an increased mortality risk in patients with rheumatoid arthritis who have higher RAR levels [9]. A historical cohort study based on the Medical Information Mart for Intensive Care III database shows that an RAR ≥ 5.51 %dL/g is independently associated with increased all‐cause mortality at 30 days, 90 days, and 1 year in patients with cancer [10]. After controlling for confounders, the hazard ratio for 30‐day mortality was 1.74 (95% CI: 1.48–2.04) in the high‐RAR group compared with the low‐RAR group. Another study involving intensive care unit patients with acute myocardial infarction finds that elevated RAR is independently associated with increased in‐hospital all‐cause mortality [11]. In patients undergoing surgery for burns, an RAR > 6.8 %dL/g on the first postoperative day is significantly (*p* < 0.001) associated with higher 90‐day mortality compared with those with RAR ≤ 6.8 %dL/g (49.2% vs. 12.3%) [12]. A retrospective observational study of patients with sepsis shows that higher RAR levels are associated with increased 28‐ and 90‐day mortality rates [13]; the odds of death in patients in the highest RAR quartile are more than four times higher than those in the lowest quartile. Other studies have also reported that higher RAR is linked to poor prognosis in patients with sepsis or acute respiratory distress syndrome [14, 15]. Recent evidence also supports the prognostic value of RAR in acute inflammatory conditions. A meta‐analysis of patients with acute pancreatitis demonstrated that elevated RAR was associated with increased mortality risk (relative risk of 2.11, 95% CI: 1.35–3.30) and greater disease severity, with significantly higher RAR values observed in severe compared with mild cases [16]. In a recent study, RAR demonstrated excellent discrimination (area under the receiver operating characteristic curve of 0.955) for in‐hospital mortality among patients undergoing surgery for scrotal Fournier′s gangrene [17]. These findings further highlight the potential utility of RAR as an early marker of systemic inflammation and clinical deterioration.

To properly interpret the results of a biomarker, it is imperative to establish its reference range, conventionally defined as the interval between the 2.5th and 97.5th percentiles of the biomarker′s distribution in an apparently healthy population [18]. To date, no study has systematically determined a population‐based reference range for RAR. However, the reference range for a biomarker may vary from population to population and by sex, age, and race. The present study was thus conducted to determine the reference interval for RAR in a large representative sample of the US population stratified by sex, age, and race.

## 2. Materials and Methods

### 2.1. Source of Data

The US Centers for Disease Control and Prevention (CDC) has established a comprehensive program that utilizes a multifaceted approach to assess the health and nutritional status of the US population. The program includes interviews, physical examinations, and laboratory tests. These data collectively provide a comprehensive dataset on the health status of the population and are stored in the NHANES database. To obtain nationally representative estimates, data from seven biennial cycles (2005–2018) of NHANES were selected and combined to extract RDW, serum albumin, and relevant demographic and health‐related variables. The data are publicly available at https://wwwn.cdc.gov/Nchs/Nhanes/.

### 2.2. Inclusion/Exclusion Criteria

Participants aged 18 years or older with available measurements of RDW and serum albumin were included in the analysis. Individuals were excluded if they were pregnant or lactating, or if they reported a history of diabetes mellitus, cardiovascular or cerebrovascular disease, liver disease, thyroid disease, kidney disease, or malignancy.

The “apparently healthy” population was defined based on the self‐reported absence of these major chronic conditions. Laboratory markers of subclinical inflammation (e.g., C‐reactive protein) were not used as exclusion criteria due to incomplete availability across NHANES cycles. Likewise, additional exclusions based on laboratory abnormalities (e.g., anemia, iron deficiency, or inflammatory markers) were not systematically applied.

### 2.3. Statistical Analysis

*R* software Version 4.6.0 (*R* Project for Statistical Computing) was used for data analysis. The continuous variables were presented as median and interquartile range (IQR); the categorical variables, absolute number, and percentage. NHANES data were collected using a multistage, stratified, and clustered sampling design. Accordingly, appropriate weights were applied to adjust for unequal probabilities of selection and nonresponse, ensuring valid results representative of the population. For this purpose, the *survey* package was utilized. The reference interval for RAR was defined as the interval between the 2.5th and 97.5th percentiles of the weighted RAR distribution, estimated using a nonparametric approach with survey weights (via weighted quantiles). No explicit outlier exclusion was performed, and all available observations were retained to reflect the population distribution. Given the large sample size in each subgroup, percentile‐based estimation was considered robust. The interval was calculated for each group stratified by sex, age group, and race.

### 2.4. Ethics

NHANES is a publicly available database. Its data collection protocols were reviewed and approved by the National Center for Health Statistics (NCHS) Ethics Review Board to ensure compliance with ethical standards and the protection of participants′ rights. Written informed consent was obtained from all NHANES participants. All data provided by NHANES were de‐identified and are classified as nonhuman subjects research, thereby exempting the current analysis from additional institutional review board approval.

## 3. Results

### 3.1. Study Population Characteristics

After applying the inclusion/exclusion criteria, the final study population comprised 11,683 men and 10,899 women. When accounting for sampling weights, these participants represented 74,047,470 men and 67,220,923 women in the US adult population (Table 1).

**Table 1:** Distribution of the basic characteristics of the study population (NHANES data, 2005–2018).

Parameter	Unweighted values			Weighted values		
	Men	Women	Total	Men	Women	Total
Number of participants	11,683 (51.7%)	10,899 (48.3%)	22,582 (100%)	74,047,470 (52.4%)	67,220,923 (47.6%)	141,268,393 (100%)
Median age (IQR∗)	42 (30–56)	43 (31–57)	42 (31–56)	40 (29–53)	42 (30–55)	41 (30–54)
Age group (years)						
< 45	6420 (55.0%)	5896 (54.1%)	12,316 (54.5%)	43,287,038 (58.5%)	37,241,459 (55.4%)	80,528,497 (57.0%)
45–64	3798 (32.5%)	3469 (31.8%)	7267 (32.2%)	25,055,232 (33.8%)	22,447,690 (33.4%)	47,502,922 (33.6%)
≥ 65	1465 (12.5%)	1534 (14.1%)	2999 (13.3%)	5,705,200 (7.7%)	7,531,774 (11.2%)	13,236,974 (9.4%)
Race						
Hispanic	3104 (26.6%)	2984 (27.4%)	6088 (27.0%)	12,053,540 (16.3%)	10,085,564 (15.0%)	22,139,104 (15.7%)
Non‐Hispanic Black	2451 (21.0%)	2429 (22.3%)	4880 (21.6%)	7,698,097 (10.4%)	8,459,205 (12.6%)	16,157,302 (11.4%)
Non‐Hispanic White	4704 (40.3%)	4117 (37.8%)	8821 (39.1%)	48,528,625 (65.5%)	43,021,192 (64.0%)	91,549,817 (64.8%)
Other	1424 (12.2%)	1369 (12.6%)	2793 (12.4%)	5,767,208 (7.8%)	5,654,962 (8.4%)	11,422,170 (8.1%)

*Note:* Asterisk “ ^∗^” denotes interquartile range.

The median age was 40 years (IQR: 29–53) for men and 42 years (IQR: 30–55) for women. Over 60% of participants were non‐Hispanic (NH) Whites, followed by Hispanics, NH Blacks, and approximately 8% from other racial/ethnic groups (weighted estimates, Table 1). More than half of the participants were younger than 45 years, one‐third were aged 45–64, and nearly 9% were 65 or older.

### 3.2. Distribution of RDW, Albumin, and RAR

The RDW was 12.9% (IQR: 12.3–13.5), with higher and more variable values observed in women than in men. RDW increased slightly with age and was highest among NH Blacks and lowest among NH Whites (Table 2). In contrast, the median serum albumin concentration was lower in women than in men (4.2 vs. 4.4 g/dL), declined modestly with age, and was lowest in NH Blacks (Table 2).

**Table 2:** The median and interquartile range (IQR) of measured variables as well as RAR reference intervals (2.5th–97.5th percentiles) stratified by sex, age group, and race after applying sampling weights.

Parameter	RDW ^∗^(%)	Albumin (g/dL)	RAR^†^ (%dL/g)	RAR^†^ reference interval (%dL/g)
Whole population	12.9 (12.3–13.5)	4.3 (4.1–4.5)	3.0 (2.8–3.2)	2.5–4.1
Sex				
Female	12.9 (12.4–13.6)	4.2 (4.0–4.4)	3.1 (2.9–3.4)	2.5–4.4
Male	12.8 (12.3–13.4)	4.4 (4.2–4.6)	2.9 (2.7–3.1)	2.5–3.7
Age group (years)				
< 45	12.8 (12.3–13.4)	4.4 (4.1–4.6)	2.9 (2.7–3.2)	2.5–4.1
45–64	12.9 (12.4–13.5)	4.3 (4.1–4.5)	3.0 (2.8–3.3)	2.6–4.1
≥ 65	13.2 (12.6–13.8)	4.2 (4.0–4.4)	3.2 (3.0–3.4)	2.6–4.2
Race				
Hispanic	12.9 (12.4–13.6)	4.3 (4.1–4.5)	3.0 (2.8–3.3)	2.5–4.2
Non‐Hispanic Black	13.4 (12.7–14.2)	4.2 (3.9–4.4)	3.2 (3.0–3.5)	2.6–4.9
Non‐Hispanic White	12.8 (12.3–13.3)	4.3 (4.1–4.5)	3.0 (2.8–3.2)	2.5–3.9
Other	12.9 (12.3–13.5)	4.3 (4.1–4.5)	3.0 (2.8–3.2)	2.5–4.1

*Note:* Asterisk “ ^∗^” denotes red blood cell distribution width. Dagger “^†^” denotes red blood cell distribution width divided by serum albumin.

**Figure 1:**
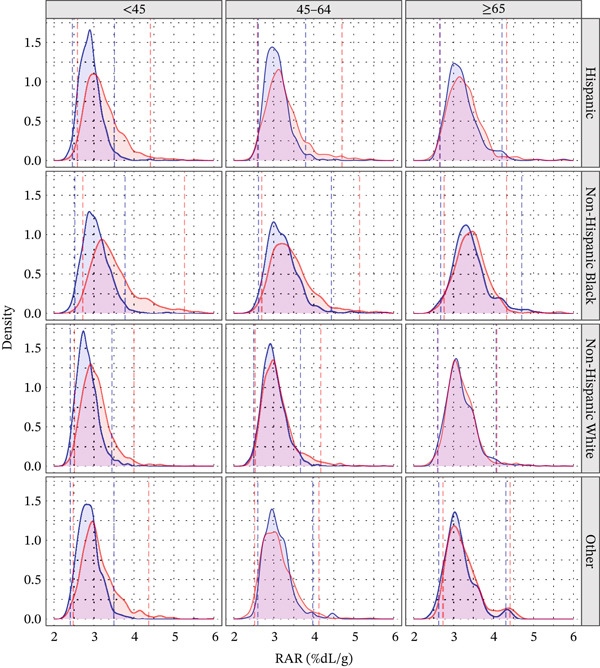
Weighted probability density distributions of RAR in US adults, stratified by sex (females, red; males, blue), race (rows), and age groups (columns). Dashed vertical lines represent the 2.5th and 97.5th percentile reference limits for each sex‐specific subgroup.

**Figure 2:**
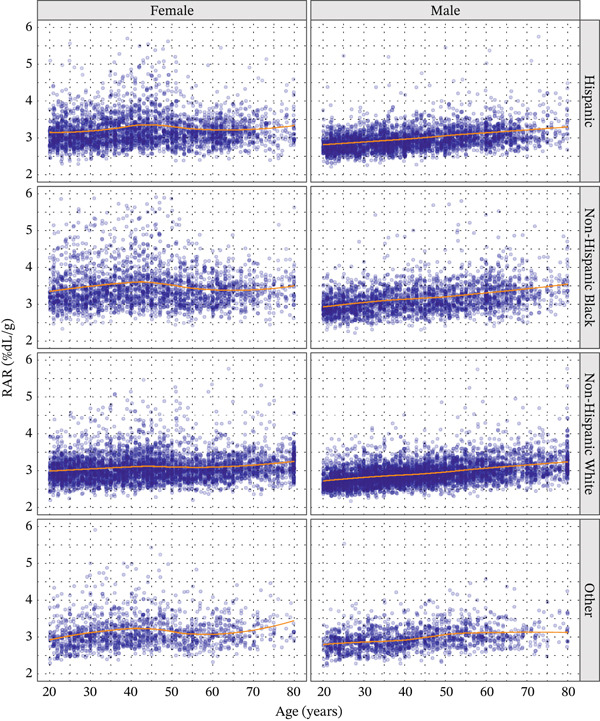
Variation of RAR with age in US adults, stratified by sex (columns) and race (rows).

The median RAR was 3.0 %dL/g (IQR: 2.8–3.2). RAR was consistently slightly higher in women than in men (Table 2; Figure 1) and exhibited a small age‐dependent increase (Figure 2). NH Blacks had marginally higher RAR values compared with other racial/ethnic groups (Table 2), along with slightly greater variability.

### 3.3. Reference Intervals for RAR

The overall reference interval for RAR was 2.5–4.1 %dL/g. Although the lower limit of the reference range remained stable across demographic strata (Table 2; Figure 1), the upper limit varied. The reference interval was generally wider in women than in men, with the widest interval observed among NH Black women under 65 years of age and the narrowest among Hispanic and NH White men in the same age group (Figure 1).

## 4. Discussion

RDW is an inexpensive and readily measurable hematologic index that reflects variability in red blood cell size. In recent years, it has garnered significant attention as a biomarker for various clinical conditions [19–21]. The findings of higher RDW values in women compared with men—likely attributable to lower iron stores secondary to blood loss due to menstruation—as well as the slight age‐related increase in RDW, align with previous reports [20, 22, 23].

Approximately 30% of RDW variability has been linked to genetic factors [19], with 194 independent genetic signals identified, 71 of which are associated with conditions including autoimmune diseases, malignancies, Parkinson′s disease, and age‐related macular degeneration. These genes are implicated in pathways related to telomere maintenance, ribosomal RNA processing, and apoptosis [19]. The greater RDW variability observed among women in our study corroborates earlier findings [20].

The lower serum albumin concentrations in women compared with men, along with the modest age‐related decline, are consistent with prior research. A large‐scale UK study analyzing over 1 million serum albumin measurements demonstrated sex‐ and age‐dependent differences, likely mediated by variations in body composition and hormonal influences, particularly estrogen [24].

Notably, RDW values are consistently higher among Black individuals compared with other racial/ethnic groups [25]. This difference may reflect a combination of factors including iron deficiency, chronic inflammation, and hematologic variations prevalent in this population, as well as genetic predisposition given that nearly 30% of RDW variability is heritable [19]. Similarly, systematic reviews indicate that Black individuals tend to have lower mean serum albumin levels than their White counterparts [26], potentially due to disparities in nutritional status and higher prevalence of conditions affecting albumin metabolism. These observations collectively explain the elevated RAR values observed among NH Blacks in the current study.

RAR, as the ratio of RDW to serum albumin, demonstrated consistent patterns: higher values in women and a gradual increase with age. This trend reflects two concurrent physiological changes—rising RDW and declining albumin levels—both of which contribute to higher RAR values. Given that elevated RDW is associated with inflammation and increased mortality risk [27, 28], whereas low serum albumin correlates with frailty and adverse outcomes [29, 30], RAR may serve as a composite marker of physiological stress with potential utility in assessing inflammation, frailty, and biological aging [8, 13]. This interpretation is consistent with recent work highlighting the clinical utility and integrative nature of composite hematologic biomarkers [31].

The reference range for RAR (2.5–4.1 %dL/g) remained relatively stable across demographic strata (Table 2, Figure 1), supporting its robustness as a biomarker. The modestly higher RAR observed among NH Blacks reflects demographic variation in RAR distribution rather than evidence of differential mortality risk, as clinical outcomes were not assessed in this study. These differences likely reflect multifactorial influences, including socioeconomic factors, chronic stress, healthcare access, and environmental exposures [32].

The stability of the lower RAR reference interval across demographic strata suggests a biological characteristic of healthy individuals. The observed variability in the upper limit may arise from several factors. Although participants with overt comorbidities were excluded from the current study, residual confounding by subclinical conditions — such as early‐stage inflammation or metabolic dysfunction — could contribute to higher RAR values in some “apparently healthy” individuals studied, thereby marginally elevating the upper limit. This effect may vary across subgroups due to differences in undiagnosed conditions or physiological stress (e.g., chronic inflammation in NH Blacks) [25, 32]. Genetic polymorphisms influencing RDW or albumin [19, 26], along with environmental factors like diet or socioeconomic stressors [32], may further modulate the upper limit of the RAR reference interval. However, the magnitude of this variability was small relative to the overall reference interval, supporting the overall consistency of RAR as a biomarker.

The study′s primary strength lies in its use of seven nationally representative NHANES biennial cycles with standardized laboratory measurements, ensuring robust estimates of RAR distributions in the general US adult population. However, the cross‐sectional design precludes causal inference, and the absence of laboratory‐based exclusions for subclinical inflammation (e.g., C‐reactive protein), anemia, or iron deficiency may have introduced residual confounding. Confidence intervals for the reference limits were not computed but may be approximated using bootstrap resampling methods. Future longitudinal studies are warranted to evaluate the predictive utility of RAR for clinical outcomes such as mortality and to further establish its role as a biomarker.

## 5. Conclusions

RAR reference intervals showed minimal variation across demographic strata, supporting the potential utility of RAR as a robust biomarker for clinical and research applications.

## Author Contributions

P.H.: conception of the idea; data collection; development of the *R* script for data analysis; data analysis and interpretation; drafting the manuscript; and substantial revision of the manuscript. D.H.: interpretation of the results and critical revision of the manuscript.

## Funding

P.H. was supported by the Thomas H. Nimick, Jr. Competitive Research Fund.

## Disclosure

Both authors approved the final version of the manuscript and agreed to be accountable for all aspects of the work.

## Conflicts of Interest

The authors declare no conflicts of interest.

## Data Availability

All relevant data are within the manuscript.
